# Provings and Clinical Observations with High Potencies

**Published:** 1893-07

**Authors:** Malcolm Macfarlan

**Affiliations:** Philadelphia


### PROVINGS AND CLINICAL OBSERVATIONS WITH HIGH POTENCIES.

MALCOLM MACFARLAN, M. D., PHILADELPHIA.

### PIX-LIQUID.$^{5C}$.

Dull headache with soreness of scalp, left side mostly affected.

### PLANTAGO-MINOR.$^{5C}$.

Extreme soreness and throbbing on top of the head; scalp so sensitive it is painful to use a comb.

### PSORIN$^{42M}$.

Aching mostly in the *back of her head.*

### PSORINUM$^{42M}$.

Severe, dull pain all over his head.

### PHYTOLACCA-DEC.$^{47M}$.

Dull pain across forehead.

### PLANTAGO-MIN.$^{11C}$.

Stiff neck, very painful on motion; aching severe; sharp pain through the head, just above the ears, leaves great soreness when it passes away; some pain in the forehead; feeling of heat in head.

### PETROL$^{CM}$.

Back and top of her head pains her; pounding sensation through the head in front of her ears and above them.

### RHUS-TOX.$^{105M}$.

Has cured many cases of *porrigo* or scald head in children, the eruption filling up the scalp and extending in some cases to the shoulders, discharge from the ears in several cases.

### SAP-SODA$^{2C}$.

Dull pain in forehead; slight aching over whole head.

### SALVIA-OFF.$^{5C}$.

*Giddiness;* aggravated by looking downward.

### SAMBUCUS-NIG.$^{45M}$.

Frontal headache with symptoms of a bad cold. It is believed that in one case, when the remedy was given for a long time, it produced symptoms similar to facial erysipelas, affecting mostly the left side.

### SARRACENIA-PURP.$^{2M}$.

*Light-headed or giddy.*

### SEPIA$^{2M}$.

Severe pains in top and back of his head.

### SECALE-CORN.$^{95M}$.

Continuous supra-orbital headache; this symptom developed after proving the remedy a week.

### SERPENTARIA$^{5C}$.

Quite dizzy and weak; feels as if she would fall at times, unless she could get hold of some object.

### SULPHUR$^{CM}$.

After trial of many remedies, Bell., Hyosc., Zinc, and Hellebore, none have had the uniformly good effect of Sulphur in relieving chronic cerebral congestion. *Brain fever of children;* vivid, frightening dreams; starting in sleep; disposition to delirium; night terrors.

### SYMPHYTUM-OFF.$^{15C}$.

General throbbing headache, all over the head; back, top, and frontal region.

### TEREBINTH$^{17M}$.

Symptoms of a common cold in the head; ringing in ears.

### TARAXACUM$^{67M}$.

Sensation of great heat on top of head.

Sterno-mastoid muscle, very painful to touch in two provers; crampy pains in different parts of the body; cramp in the left sterno-mastoid muscles; causes him to incline his head for a day or two.

### TRIFOLIUM-PRAT.$^{10M}$.

*Headache* over both eyes relieved by heat and friction.

### TELLURIUM$^{5C}$.

Quickly cured neuralgia of the side of face and head, affecting the fifth pair of nerves; often verified.

### TRIF-ALB.$^{1M}$.

Head giddy, with sense of fullness; face flushed.

### UVA-URSI$^{10M}$.

Symptoms of cold in head or general cold.

### WOORARI$^{90M}$.

Hammering sensation within the head.

### EYES.

### ALETRIS$^{45M}$.

Lids inflamed; eyes water.

### APIUM$^{69M}$.

Sensation as if sand were in the eyes; lids slightly red.

### ARSENIC$^{6M}$.

Vision blurred; eyes not much inflamed; no lachrymation; highly curative in *scrofulous ophthalmia* and ophthalmia tarsi, where the disease is communicated, particularly in children, frequently verified this. Photophobia is a marked symptom when Arsenic cures.

Given to those having conjunctivitis it produces great *intoler-*

*ance of sunlight* when photophobia was not previously a prominent symptom.

### ARUM-TRIF.$^{16M}$.

Eyes dim, water readily.

### ANTIMONY-CRUD.$^{6M}$.

Lids inflamed; water a great deal; eyelids feel as if too heavy to keep them open; left eye mostly affected; worse in morning. No disposition to read or use the eyes; intolerance of light.

### ATROPINE-SULPHATE$^{30th}$.

Gave immediate and permanent relief in several cases of ulceration of the cornea with photophobia. Severe conjunctivitis and lachrymation.

Found exceedingly useful and curative in severe eye symptoms, threatening blindness, which followed syphilis.

### BELL.$^{101M}$.

Caused a burning sensation when given to cases of scrofulous ophthalmia and inflammation of the lids, highly curative in photophobia; given to chronic cases it simplifies them and prepares the way for their speedy cure by other medicines.

### BAPTISIA-TINCT.$^{6M}$.

Eye-lids inflamed; photophobia without lacrymation.

### CAUST.$^{30M}$.

Profuse lachrymation; light and heat are hurtful.

### CINA$^{12C}$.

Twitching of eyelids; disposition to be cross-eyed.

### CALEND-OFFIC.$^{45M}$.

Aching in the eyes accompanying the headache.

### CANCER-ASTACUS$^{5C}$.

Movement of the eye causes pain.

### CANNABIS-SAT.$^{1M}$.

Causes symptoms like acute conjunctivitis.

### CORNUS-F.$^{45M}$.

Eyes weak; left mostly affected; lachrymation.

### CUPRI-ACETAS$^{45M}$.

Often highly curative in scrofulous ophthalmia of children when other remedies have been given without benefit.

### EUPAT-PERF.$^{CM}$.

Occasional intense sharp pain in the eyes as if they were being pierced.

### FLUORIC-ACID$^{45M}$.

Pain in the eyeballs to a slight degree.

### GELSEM-NIT.$^{CM}$.

Eyes water and slightly inflamed; heaviness of the lids.

### GRAPH.$^{CM}$.

Margins of the lids inflamed; itching and soreness of the eyelids externally.

### HYPERICUM-PERF.$^{45M}$.

Glands of lids inflamed; disposition to small styes.

### KALI-HYD.$^{CM}$.

With occasionally Merc-cor., cured quickly a case of old scrofulous ophthalmia, starch poultices used nightly.

### IRIS-VERS.$^{CM}$.

Symptoms of asthenopia, with nausea; indigestion.

### IODINE$^{17M}$.

Dimness of vision; asthenopia; slight inflammation of lids.

### KALMIA-LATIFOLIA$^{12C}$.

Eyes weak and water a great deal.

### LACTUCA-VIR.$^{5C}$.

Pain in the eyelids with slight inflammation; smarting sensation externally.

### MERC-VIV.$^{101M}$.

Highly curative in many cases of chronic ophthalmia; movements of the eyeball are painful.

### MYRICA-CERIF.$^{45M}$.

Blurred vision at times; appearance as if of a flame before the eyes; inability to use the eyes well; over-sensitive retina.

### NATRUM-MUR.$^{CM}$.

One the most generally useful remedies in weakness of vision after excesses, illness, and from other causes.

### NUX-VOMICA$^{94M}$.

Has been highly curative in scrofulous ophthalmia where indigestion is the most pronounced condition; photophobia. As a rule, the left eye is more quickly helped than the right after giving this remedy.

### NITRIC-ACID$^{5C}$.

Eyes weak, water readily; eyeballs excessively painful and sore to pressure.

### OXALIS$^{1M}$.

Blurred vision, attended with excess of tears.

### PLANTAGO-MIN.$^{11C}$.

Eyes very weak, can hardly see with them, not inflamed; sensitive to light.

### PLANTAGO-MAJ.$^{5C}$.

Misty vision; eyes sensitive to light; no disposition to read.

### PSORIN.$^{42M}$.

Eyes much inflamed, left more than right; supra-orbital pain; profuse lachrymation; intolerance of light; tear sac very sensitive.

### PHOSPHORUS^CM.

Cured scintillations of light; asthenopia; symptoms frequently verified.

### PIX-LIQUIDA^5C.

Itching and burning sensation about the eyelids; fine prickling sensations come and go in the lids.

### PHYTOLACCA-DEC.^47M.

Sharp pains pass through the eyeball on looking at small objects, or straining the vision.

### PULSATILLA-PRET.^CM.

Very efficacious in curing fistula lachrymalis; thick, yellow discharge. Verified this a great many times and with a variety of potencies; a remarkable remedy in this respect.

### RANUNCULUS-SCLER.^2M.

Eyes very weak and water much; lids greatly inflamed.

### RANUNCULUS-ACRIS^10M.

Eyes itchy and light affects them severely; symptoms of catarrhal ophthalmia.

### RHUS-TOX.^105M.

Slight redness of the conjunctiva. Rhus is probably the most curative medicine in severe ciliary pain. A most valuable remedy to subdue inflammatory symptoms following cataract operations or iridectomy. It has often acted like magic. Useful in relieving the pain in glaucoma.

### SAP-SODA^2C.

Dull, heavy pain across the eyes; eyes weak, remarkably so, not inflamed, simply tired or weak.

### SANTONINE^6 dec,.

Cured a great many cases of ulcer of cornea; intolerance of light. Old, obstinate cases of chronic ophthalmia in young children ; constant lachrymation. The cases do better with a few preliminary doses of Sulphur.

### SARRACENIA-PURP.$^{2M}$.

Slight conjunctivitis ; intolerance of light.

### SEPIA$^{45M}$.

Produced marked conjunctivitis, which quickly passed away on stopping the medicine.

### SECALE-CORN.$^{95M}$.

Pain from forehead to the eyes ; burning sensation in the eyes ; no inflammation ; pain shoots through the eyeball backward.

### SPIGELIA$^{75C}$.

Frequently cured involuntary movements of the eyeballs and twitching of eyelids ; disposition to squint.

### SULPHUR$^{CM}$.

Highly curative in scrofulous inflammation of lids ; disposition to facial eczema, in children ; it cured two severe cases of amblyopia, one a girl, the other a man ; curative in some cases of diplopia. A great deal of precious time was wasted for patients in finding out that this is one of the most valuable remedies in many forms of chronic inflammation of the lids and cornea.

### SULPHATE OF MORPHIA$^{6\ dec}$.

Given every hour for two weeks, produced on third day violent pain through the eyeballs ; dimness of sight ; pupils not dilated, retina undersensitive ; had previously good sight, but could not read ; type became blurred, even when holding reading-matter at the usual proper distance vision was blurred and indistinct. Throat dry and parched ; nausea ; fullness in the forehead ; husky voice.

### SPONGIA-TOST.$^{CM}$.

Indistinct and blurred vision because of lid symptoms.

### Symphytum-off.$^{15C}$.

Itching of eyelids, disposition to rub them.

### Tart-emet.$^{54M}$.

Severe inflammation of conjunctiva ; profuse lachrymation.

### Triticum-rep.$^{5C}$.

Itching of eyelids, with more or less burning and stinging sensation.

### Terebinth$^{17M}$.

Produced conjunctivitis in a mild form.

### Trifolium-pret.$^{10M}$.

Eyes watery ; described as weak by prover.

### Zinc-sulph.$^{5C}$.

Curative in chronic conjunctivitis with photophobia. The most remarkable remedy in curing nearly every variety of inflammation of the cornea, whether acute or chronic. Its action is often immediate and permanent.

### EARS.

### Arsen-acid$^{6M}$.

Irritation in the meatus; itching, with slight discharge.

### Apium$^{69M}$.

Pain in the inner ear, aggravated by using the jaws.

### Ailanthus$^{45M}$.

Ears very hot, red, itchy, and swollen; worse at night.

### Bursa-past.$^{9M}$.

Slight deafness and pain in left ear.

### Calendula-off.$^{45M}$.

Left ear ached much, followed by discharge.

### Cochlearia-a.$^{10M}$.

Pain in left ear, deep internal aching; worse when she stoops or comes near the range.

### Gelsem-nit.$^{CM}$.

Left ear discharges freely, which it never did before.

### Ginseng$^{46M}$.

Ringing in his ears when he had pain in his head.

### Gummi-gutti$^{5C}$.

Left ear ringing continually; sometimes hissing.

### Mentha-pip.$^{5C}$.

Soreness in ears.

### Nat-mur.$^{CM}$.

Caused buzzing in ears.

### Nitrum$^{5C}$.

Spasmodic clicking in the ears, like opening and shutting, clicking sensation, very annoying.

### Psorin.

Own voice sounds unnatural; sounding and humming in left ear, as from a sea-shell.

### Psorin$^{42M}$.

A most wonderful medicine, has cured many times offensive discharge from the ears (acute and chronic) for me. Cured scabby eczema behind ears in infants, with deafness of long standing. Often cured *old discharge from* ears in adults.

### Psorinum$^{42M}$.

Pain in his ear so great as to confine him to bed for five days. External ear much swollen, thought the pain in his ear would set him crazy. Soreness in jaw, right side, around the ear; could not open his mouth because of the contraction and pain; could scarcely crowd his fingers between his teeth.

### SILICEA$^{CM}$.

Caused great earache with some discharge, never had it before.

### SULPHUR$^{CM}$.

Cured slight offensive discharges from the ear in several young persons.

### PHYTOLACCA-DEC.$^{47M}$.

*Swelling* in and around the left ear and side of the face like erysipelas, then ran over the scalp, very painful to touch.

### SAMBUCUS-NIG.$^{45M}$.

Great swelling, heat, and redness in the neck, just under the right ear, with sharp pain; improved his hearing, which had been defective; restless at night.

### TEREBINTH.$^{17M}$.

Own voice sounds unnatural; sounding and humming in left ear as if from a sea shell; cannot tell where the person is who speaks unless he sees him; talking loud is very painful.

### NOSE.

### ARSENIC-ALB.$^{72}$.

Watery discharge from the nose.

### AGARICUS-MUSC.$^{2M}$.

Pimples appear on side of her nose.

### ARUM-TRIF.$^{16M}$.

Thick discharge from high up opposite the nasal bone on right side. Continually picking his nose because of irritation within.

### BURSA-PAST.$^{9M}$.

Free discharge of blood and mucus, mostly from left nostril.

### Camphor$^{30M}$.

Given five times daily caused hemorrhage from the nose on fourth day; hemorrhage *every* day for two weeks, and still went on for some time after stopping medicine; weakened him very much; never had nose-bleed before taking this medicine. Produced nose-bleed in other provers.

### Ginseng$^{46M}$.

Frequently blowing his nose.

### Hydrastis-can.$^{CM}$.

Nose discharges freely.

### Hypericum-perf.$^{45M}$.

Sore within the nose; itchy; continually picking it; observed in all the provers. A valuable and reliable symptom.

### Iodine$^{17M}$.

Constant watery discharge.

### Kali-hyd.$^{CM}$.

Produced sores in and about the nostrils, with discharge.

### Kaolin$^{45}$.

Uses six or eight handkerchiefs a day, discharge so abundant.

### Nux-vom.$^{94M}$.

Occasional watery discharge from nose.

### Oxalis$^{1M}$.

Her nose bled twice and has been very sore inside; has not bled for years before. One prover.

### Phosphorus$^{CM}$.

Stuffiness of nose; sniffles.

### PSORIN$^{42M}$.

The pains extend from eyebrows over the nose, and caused the nose to discharge somewhat.

### RANUNCULUS-ACRIS$^{10M}$.

Stuffed up feeling in nose.

### PIX-LIQUIDA$^{5C}$.

Dryness and burning at nasal bones.

### TROMBIDIUM$^{5C}$.

Dryness in nose, symptoms of sniffles.

### TRITICUM$^{15C}$.

Always blowing her nose; nose sore to touch.

### SANTONINE$^{3rd}$.

Given every hour on and after the third day caused constant sneezing; discharge from nose; sore nose.

### SYMPHYTUM-OFFIC.$^{15C}$.

Nose sore inside the alae; wants to pick it.

### UVA-URSI$^{10M}$.

Nose is raw and discharges.

### FACE.

### ÆSCULUS-HIP.$^{56M}$.

Pimples appeared on his face and body; hands and face swell up greatly and become red after washing.

### ARGENT-NIT.$^{45M}$.

Left side of the face appears swollen, with a great deal of heat and burning; lips much swollen.

### ASTERIAS-RUB.$^{5C}$.

Pimples on the side of nose and chin (female provers).

AILANTHUS$^{45M}$.

A crop of ragged, deep ulcers on the lower lip and corner of the mouth; the least touch or pressure aggravates this; face speckled. Cured acne on a girl's face.

ARSENIC-ALB.$^{6M}$.

Face swelled.

BELL.$^{101M}$.

Caused a feeling in cheek-bones as if scalded when given in old cases of ophthalmia. Swollen cheeks and lips.

CUPRUM-ACET.$^{45M}$.

Sharp pain; sore to touch in both malar bones.

EUPAT-PERF.$^{CM}$.

Sudden violent contraction of muscles of right cheek.

FERRI-CARB.$^{50M}$.

Face suddenly livid, red, or purple, then pale, rest of body cold, then legs red and head pale and cold, or fever with redness. Flushes coming and going rapidly in various parts of body.

GUAJACUM$^{5C}$.

Her face became spotted after fever commenced; eyes, nose, and cheeks would swell.

IODINE$^{17M}$.

Face fiery red.

KREOSOTE$^{CM}$.

Produced pimply rash over the face like flea bites.

NITRUM$^{5C}$.

While eating she would bite her lips involuntarily, this continued while she took the medicine.

NITRIC-ACID$^{5C}$.

Rash on her face and forehead, small pimples.

PLANTAGO-MIN.$^{11M}$.

Redness of the skin (like erysipelas) in the right temple mostly.

POLYGONUM-HYDROP.$^{45M}$.

Sore pimples on face.

RANUNCULUS-ACRIS$^{10M}$.

Burning in face.

SAP-SODA$^{2C}$.

Face looks swollen.

VERBASCUM-THAP.$^{45M}$.

Pain in both cheek bones, and above her eyebrows.

WOORARI$^{90M}$.

Her lips become very blue; circulation irregular.

### TEETH.

ASTER-RUB.$^{5C}$.

Neuralgia opposite the left molar teeth, lower jaw; sharp, piercing pain like a needle.

BURSA-PAST.$^{9M}$.

Teeth are sore when she shuts down on them; gums are sore, she has not toothache, but neuralgic feeling in her teeth.

DIGITALIS-PURP.$^{CM}$.

Severe toothache, lasting three days; pain through her whole head accompanied it.

IODINE$^{17C}$.

Teeth sticky with phlegm.

PLANTAGO-MIN.$^{11M}$.

For a moment or two her jaws become closed, and she has trouble to open her mouth; pains through her teeth.

PIX-LIQUIDA$^{4C}$.

Bad teeth more sensitive than usual.

RHODODENDRON-CHRYS.$^{CM}$.

Causes toothache and swollen gums; effected an astonishing and immediate cure of neuralgia of the inferior and superior dental nerves; woman in agony, slept only at short intervals for seven weeks; had sound molars removed and narcotics given by her regular physician without curative effect; often verified its curative powers in certain toothaches; causes stiffness of neck and stumps of teeth to be loosened.

RHUS$^{105M}$.

Teeth become loose a little.

SASSAFRAS$^{5C}$.

Toothache of a mild type.

TRIF-PRETENS$^{10M}$.

Shooting neuralgic pain in the teeth.

### MOUTH.

ALETRIS$^{45M}$.

Spits very much; saliva excessive.

APOCYNUM$^{6M}$.

Tongue heavily coated, brownish white.

AGARIC$^{2M}$.

Eruption about her lips.

ARSENIC$^{6M}$.

Mucous membrane of mouth much swollen.

ARUM-TRIF.$^{10M}$.

End of her tongue feels very sore, as if scalded; sores on tongue.

ARUM-TRIF.$^{16M}$.

Mouth very dry; feels as if the skin of the mouth would crack, so dry; wants to wet it, not to drink; has to get up in the night to do this.

BELL.$^{101M}$.

Tongue thickly coated; aching pain in all the teeth, left side mostly; front teeth felt sore to the touch. Medicine was given five times a day for three weeks.

BURSA-PAST.$^{9M}$.

Probably caused great enlargement of Wharton's duct. Lips sore. Gums felt as if blistered, inner surface and left side mostly affected.

CUPRUM-ACET.$^{45M}$.

Tongue coated with something which seems to her like pus, offensive taste.

MERC-CORR.$^{5C}$.

Sensation as if the mouth was scalded.

MERC-VIV.$^{101M}$.

Cured chronic tendency to hawk and spit; much previous treatment ineffectual.

NATRUM-MUR.$^{CM}$.

Fever blisters on lips, caused sores in mouth.

NITRIC-ACID$^{5C}$.

Sores all around the mouth like fever blisters; *gums and margin of the mouth covered with sores;* nostrils affected.

NUX-VOM.$^{94M}$.

Sore patches on mouth and tongue; coated tongue; dislikes his usual tobacco. Often cleared off rapidly a thickly-coated tongue.

PHYTOLACCA-DEC.$^{47M}$.

*Sores on tongue,* inside the mouth and lips.

PIX-LIQUIDA$^{5C}$.

Gums sore.

PLANTAGO-MIN.$^{11C}$.

Dry tongue.

PODOPHYL-PELT.$^{7M}$.

Continually coated tongue became perfectly red and clean; removed a breath which was foul-smelling.

RANUNCULUS-ACRIS$^{10M}$.

Mouth sore; sensation of lump in her throat, which she cannot dislodge.

RHUS-TOX.$^{105M}$.

Mouth sore; apthæ.

SAMBUCUS-NIG.$^{45M}$.

Tongue sore, mostly in middle, and very much coated.

SANGUINARIA$^{24M}$.

Sores around the gums and on roof of mouth.

WOORARI$^{90M}$.

Very offensive taste in the mouth.

ZINC-SULPH.$^{5C}$.

Mouth felt and was sore; tongue slightly sore and felt thick.

ZINC-MET.$^{2C}$.

Mouth and lips very sore; tongue affected, very sore in spots as large as the small finger-nail.

